# First record of native entomopathogenic nematodes from Montana agroecosystems

**DOI:** 10.21307/jofnem-2020-060

**Published:** 2020-07-06

**Authors:** Ramandeep K. Sandhi, Ratnasri Pothula, Satyendra K. Pothula, Byron J. Adams, Gadi V.P. Reddy

**Affiliations:** 1 Department of Research Centers, Western Triangle Agricultural Research Center, Montana State University, 9546 Old Shelby Rd., P.O. Box 656, Conrad, MT 59425; 2 Department of Biology, Monte L. Bean Museum, and Evolutionary Ecology Laboratories, Brigham Young University, Provo, UT 84602; 3 USDA-ARS, Southern Insect Management Research Unit, 141 Experiment Station Road, P.O. Box 346, Stoneville, MS 38776

**Keywords:** Entomopathogenic nematodes, Golden Triangle Region of Montana, Heterorhabditis, Steinernema

## Abstract

A total of 30 different agricultural fields in the Golden Triangle Region of Montana, USA were surveyed, and 150 soil samples were evaluated for the presence of entomopathogenic nematodes (EPNs). The authors isolated EPNs from 10% of the collected samples. The recovered isolates were identified as *Steinernema feltiae* and *Heterorhabditis bacteriophora* by using morphological and molecular analysis. *Steinernema feltiae* was found from two fields, Kalispell (*S. feltiae* 1) and Choteau (*S. feltiae* 2). *Steinernema feltiae* (1 and 2) differed significantly from each other in terms of morphological characters for infective juveniles (distance from anterior end to excretory pore and nerve ring) and 1st generation males (body length, spicule length, gubernaculum length, oesophagus, tail, and anal body diameter). *Steinernema feltiae* 2 and *H. bacteriophora* were recovered from the same field in Choteau. All these species were recovered from wheat fields with sandy clay loam and loam soils with 3.3 to 3.4% organic matter content and pH 8.

Entomopathogenic nematodes (EPNs), which occur naturally in soils, are obligate parasites of soil-inhabiting insects. EPNs were first described in 1923 with the identification of *Aplectana kraussei* Steiner (now known as *Steinernema kraussei*) (Nguyen and Hunt, 2007). Steinernematidae and Heterorhabditidae are two major families of EPNs with potential for managing insect populations (Kaya and Gaugler, 1993; Georgis et al., 2006). EPNs are associated with endosymbiotic bacteria belonging to the genera *Xenorhabdus* Thomas and Poinar and *Photorhabdus* Boemare, Akhurst, and Mourant, respectively (Boemare et al., 1993). EPNs, in association with their bacterial symbionts, are able to kill a wide range of insect hosts, usually within 24 to 48 hr. EPNs penetrate the insect host body and release symbiotic bacteria, causing septicemia that ultimately kills the host.

EPNs are widely distributed throughout the world and have been reported from different kinds of natural and managed habitats and a wide variety of soils (Hominick, 2002; Adams et al., 2006; Adams et al., 2007). The only continent where they have not been found is Antarctica (Griffin et al., 1990). About 95 species of *Steinernema* and 16 species of *Heterorhabditis* have been described so far (Hunt and Nguyen, 2016). However, there are reports of new EPN species being found and described from different parts of the world (Lephoto and Gray, 2019; Stock et al., 2019; Katumanyane et al., 2020; Půža et al., 2020). The distribution and abundance of EPNs varies depending upon the season, habitat, and geographic region. The presence and survival of EPNs is affected by various factors, especially soil texture, moisture content, temperature, and host availability (Shapiro-Ilan et al., 2012; Stuart et al., 2015).

The rapid killing of the insect host by EPNs and their feasibility of mass production (Ehlers, 2001) has increased interest in searching for and using EPNs in integrated pest management systems (Georgis et al., 2006). Therefore, search for additional potential EPN species is being carried out in different areas on a regular basis (Cimen et al., 2016; Malan et al. 2016; Majić et al., 2018; Godjo et al., 2019; Stock et al., 2019). Indigenous EPNs have been used successfully as biological control agents to suppress various insect populations (Shapiro-Ilan et al., 2002). Indigenous EPNs are more suitable for inundative application against insect pests because of their better adaptation to local environmental conditions, allowing for more persistence and thus greater biological control efficacy. Such native nematodes can be developed as new biological control agents against important insect pests (Burnell and Stock, 2000; Grewal et al., 2002; Lewis et al., 2006). Some previous surveys have focused on finding new EPN species to control important agricultural and horticultural pests under specific conditions (Campos-Herrera and Gutiérrez, 2009). Native EPN species have been isolated from different areas that have showed better heat tolerance (Solomon et al., 2000), foraging ability, virulence (Yu et al., 2010), reproductive potential, or cold adaptation (Ivanova et al., 2001). In addition, the use of exotic EPNs can result in the suppression of native nematodes (Duncan et al., 2003).

Montana is the fourth largest state in the United States with a wide range of habitats, and it can potentially harbor an equally diverse group of EPNs. Until now, no organized survey has been conducted to locate EPNs in this area. Therefore, the objective of this study was to survey EPN diversity in a variety of agricultural habitats in the Golden Triangle Region of Montana and to isolate and identify these EPNs.

## Materials and methods

### Site description and soil sampling

Montana is a landlocked state in the northwestern United States and its economy is primarily based on agriculture, including ranching and production of grain (www.mt.gov). The Golden Triangle is situated in north central Montana (N46.965260, W109.533691). Average annual precipitation in this region is 380 mm. Mean daytime temperatures range from −2.2°C in January to 29.2°C in July (Montana Office of Tourism, FAQ 2013). Annual relative humidity varies from 75.5 to 81.7%. Annual snowfall varies from 300 inches (7620 mm) in some parts of the mountains in the western half of the state, to around 20 inches (508 mm) at some locations northeast of the Continental Divide. The Golden Triangle region includes the area north of Great Falls through Cut Bank to the south of the Canadian border and from Great Falls through Havre to the Canadian border. The three points of this triangle in north-central Montana are Havre, Conrad, and Great Falls. This region is named for its good dryland wheat-growing conditions.

In the summer of 2018 (May to September), a survey was conducted through parts of the Golden Triangle region. The areas covered during the survey are shown in Table 1. The survey was mainly orientated toward 30 cultivated fields in the region including wheat, lentils, chickpea, peas, alfalfa, and fallow without any crops (Table 1) covering almost all the crops grown in the region. In all the fields, five random 10 to 15 cm deep soil samples were taken within each of the five random plots (8-10 m^2^) with the help of a hand shovel. Between samples, the shovel was thoroughly rinsed with 70% ethanol to prevent further contamination. Five random samples from each plot were combined to make one composite sample, providing five composite samples from each field. Overall, there were 150 composite samples from 30 fields. The collected samples were then placed in polyethylene bags to prevent water loss and kept in coolers (10°C) during transit to the laboratory. At each field site, data on sampling location, habitat (vegetation), longitude, latitude and elevation were recorded. For each sampling site, a subsample (*ca* 300 g) was analyzed for the physical and chemical characteristics: pH, organic matter, sand, silt, and clay content (Agvise laboratories, North Dakota) (Table 1).

**Table 1:** Different agricultural field sites surveyed in Montana, USA, for native entomopathogenic nematodes during 2018.

Sampling site	Farmer	Latitude	Longitude	Elevation (m)	Vegetation	Soil texture	Soil pH	Organic Matter (%)	Presence/Absence of EPNs	Re-culture^*^
Pendroy	John Stoltz	N48.04130°	W112.16945°	4220	Wheat/Canola	Clay loam	8.2	3.9	+(3)	No
Pendroy	Kevin Johnson	N48.04206°	W112.20099°	4328	Wheat	Sandy clay loam	7.9	4.4	+(1)	No
Choteau	Joe Miller	N47.56785°	W112.13926°	3906	Fallow	Clay	7.8	3.2	**−**	**−**
Choteau	Mike Leys	N47.90238°	W112.3802°	**−**	Wheat	Sandy clay loam	8	3.3	+(2)	Yes (1 Steinernematid and 1 Heterorhabditid)
Valier	John Majerus	N48.18454°	W111.55524°	**−**	Wheat	Sandy clay loam	8.1	3.4	**−**	
Conrad	Mark Grub	N48.6334°	W111.53175°	**−**	Peas	Sandy clay loam	7	2.9	**−**	**−**
Conrad	Mark Orcutt	N48.16259°	W111.52896°	3498	Fallow	Clay loam	8.1	2.3	**−**	**−**
Conrad	Garett Grub	N47.56786°	W112.13930°	**−**	Wheat/Vegetables	Sandy clay loam	7.8	2.4	+(2)	No
Kalispell	Ron De Yong	N48.1945°	W114.2341°	**−**	Fallow/Winter wheat	Loam	8.1	3.4	+(1)	Yes (1 Steinernematid)
Valier	Jeremy Curry	N48.3032°	W112.4055°	3749	Fallow/Wheat	Sandy clay loam	8.2	2.1	+(2)	No
Conrad	Zane Drishinski	N48.77764°	W111.8948°	3508	Alfalfa	Clay loam	6.8	2.8	**−**	**−**
Conrad	WTARC	N48.3076°	W111.9255°	**−**	Fallow/Wheat	Clay loam	7.8	2.9	**−**	**−**
Conrad	Garman	N48.27808°	W111.92616°	1116.05	Alfalfa	Clay loam	8.1	3.7	**−**	**−**
Choteau (Knees)	A Killion	N48.00729°	W111.36596°	1081.26	Chickpea	Clay loam	8.2	2.5	**−**	**−**
Knees	A Killion	N48.00729°	W111.36596°	1081.26	Flax	Clay loam	7.5	3.5	+(1)	No
Brady	Winterhood	N47.98468°	W111.84313°	1112.80	Fallow/Winter wheat	Sandy clay loam	7.3	1.9	+(1)	No
Collins	Chickenhead	N47.95588°	W111.90971°	1122.68	Chickpea	Clay	7.8	2.3	+(1)	No
Choteau		N47.91965°	W112.04917°	1177.36	Hemp	Clay	8.2	2.8	**−**	**−**
Choteau	Nesbitt	N47.79007°	W111.16589°	1224.32	Durum Wheat	Clay loam	6.6	4.4	**−**	**−**
Dutton	Tims	N47.83529°	W111.53990°	1144.20	Lentils	Clay loam	7.9	3.4	**−**	**−**
Shelby west	John Wigand	N48.56643°	W111.98537°	1103.57	Peas	Sandy clay loam	6.9	3.9	**−**	**−**
Shelby west	John Wigand	N48.57870°	W111.99317°	1087.12	Triticale	Sandy loam	7.6	2.6	**−**	**−**
Shelby West	John Wigand	N48.54374°	W111.99096°	1071.82	Fallow	Clay loam	7.6	2.3	**−**	**−**
Potter Rd Shelby	John Wigand	N48.65339°	W111.96044°	1057.40	Fallow	Clay loam	7.9	2.7	**−**	**−**
Sunburst	Koeye Fauque	N48.82692°	W111.85641°	1093.77	Chickpea	Sandy clay loam	8	2.9	+(1)	No
Sunburst	Koeye Fauque	N48.82701°	W111.81002°	1102.46	Spring wheat	Clay loam	8.2	2.3	**−**	**−**
Tiber	Duncan	N48.19011°	W111.25206°	993.03	Fallow	Clay loam	8.2	2.6	**−**	**−**
Tiber	Paul Kolstad	N48.17184°	W111.17372°	960.91	Fallow	Clay loam	8.1	2.3	**−**	**−**
Tiber	Wicks	N48.23188°	W111.14511°	1029.37	Lentil/Fallow	Clay loam	7.7	2.5	**−**	**−**
Tiber	Broadhurst	N48.27280°	W111.14571°	930.70	Fallow	Clay loam	8.1	2.6	**−**	**−**

**Notes:** The number in the parenthesis represents the number of positive samples with nematodes. ^*^Reculture means if we were able to reculture EPN isolates found from the samples in *G. mellonella*

### Nematode isolation

EPNs were isolated from the soil samples using insect baiting techniques (Bedding and Akhurst, 1975). Within a week of soil sampling, a 300 g subsample was transferred to a 500 ml plastic container. Five larvae of *Galleria mellonella* L. (Lepidoptera: Pyralidae) were added as bait into each cup. The containers were kept in the dark at room temperature (22±2°C). After five to six days of incubation, the dead larvae were removed and rinsed with tap water. The dead larvae that exhibited signs of possible infection with EPNs (e.g., flacid, soft, odorless larvae that were dark brown, orange, dark red, pale yellowish, brown, or black in color) were washed and placed in modified White traps (White, 1927). The larvae on the White traps were checked after one week and daily thereafter for emergence of infective juveniles (IJs). All IJs emerging from cadavers from a given soil sample were collected and considered as one isolate. In the case of negative results, the isolation process was repeated once to confirm results of the first isolation following the same procedure. To confirm the virulence of the collected nematodes and to establish new cultures, collected IJs were used to infect fresh *G. mellonella* larvae (Kaya and Stock, 1997). Dead larvae were collected and placed on White traps. The emerged IJs were collected in distilled water for 10 days and stored in tissue culture flasks at 10°C. These nematodes were re-cultured monthly.

### Morphological characterization

The extracted IJs from different fields were cultured on last instar larvae of *G. mellonella.* Ten *G. mellonella* larvae were inoculated with 1,000 to 2,000 IJs (100-200 IJs/larva) for each isolate in 9 cm-diameter Petri dishes. These Petri dishes were observed for nematode infection daily. Infected cadavers were dissected in Ringer’s solution according to the procedure of Kaya and Stock (1997). Overall, 20 males and IJs were observed for all the test isolates. The adults and IJs were killed in hot water at 60°C and fixed in an equal parts mix of hot TAF (60°C) and Ringer’s solution. The nematodes were left in the fixative for two days, after which they were processed through glycerin in a vacuum desiccator as explained by Kaya and Stock (1997).

The processed males and IJs were mounted on glass slides and observed for different morphological characters: body length (L), maximum body width (D), tail length (TL), anal body width (ABD), distance from the anterior end to oesophagus (ES), distance from anterior end to excretory pore (EP), and distance from anterior end to nerve ring (NR). In addition, males were observed for spicule length (SP) and Gubernaculum length (GU). The different morphological measurements were recorded using ToupView 3.7 software (Zhejiang, China). According to their morphology, all isolates were placed into different species groups using taxonomic criteria as suggested by Hominick et al. (1997). The morphological characteristics of IJs and males of the EPN isolates were compared statistically by using two-sided *t*-test (*α*=0.05) in R 3.5.2 (R Development Core Team, 2020).

### Molecular characterization

For DNA extraction, pooled EPN IJs of each isolate were macerated with a plastic pestle in 1.5 ml centrifuge tube and genomic DNA was extracted using Qiagen DNeasy® Blood and Tissue kit (Waltham, MA) by following manufacturer’s protocol (Qiagen DNeasy® Blood & Tissue Handbook, 2006). The extracted DNA was concentrated to 20 μl using Eppendorf Vacufuge Plus Vacuum Concentrator (Hamburg, Germany). A part of rDNA comprising the internal transcribed spacer regions (ITS), ITS1 and ITS2 including 5.8 S were sequenced using two sets of primers. Primer set ITS-F (5’-TTGAACCGGGTAAAAGTCG-3 and ITS-R (5’-TTAGTTTCTTTTCCTCCGCT-3’) was used to sequence the entire ITS1, 5.8 S and ITS2 regions (Nadler et al., 2000) while primer set Fnema18S (5’-TTGATTACGTCCCTGCCCTTT-3’) and rDNA1.58 S (rev) (5’-ACGAGCCGAGTGATCCACCG-3’) pair targeted the ITS1 region (Cherry et al., 1997). Each PCR reaction was carried out in a total volume of 30 μl consisting of 9 μl of DNA template, 15 μl of JumpStart™ REDTaq® ReadyMix (Sigma-Aldrich, St. Louis, MO), 2.4 μl of each primer and 1.2 μl of molecular grade water. The PCR conditions included initial denaturation at 94°C for 5 min, 40 cycles of denaturation at 94°C for 30 sec, 40 cycles of annealing at 48°C for 30 sec, 40 cycles of extension at 0.5°C/sec for 90 sec and a final extension at 72°C for 5 min. The PCR products were analyzed for expected DNA band weights on 1% agarose gel run at 150 V for 20 min. PCR products were treated with ExoSAP-IT™ PCR Product Cleanup Reagent (Applied Biosystems, Foster City, CA) according to the manufacturer’s protocol to digest excess primers and nucleotides. The products were sequenced bidirectionally with their PCR primers using Bigdye reaction chemistry on an ABI ABI3730xl. Primer sequences were removed from chromatograms and sequences were aligned and edited manually in Geneious Prime 2019.2.1 (http://www.geneious.com). Each species was identified via BLASTn (NCBI; http://www.ncbi.nlm.nih.gov) against the nucleotide collection (nr/nt) database using default search parameters.

## Results

Overall, 150 composite samples (750 single point samples) were collected from the 30 fields in the survey area. Nematodes were recovered from only 15 of the 150 samples. The total percent nematode recovery from samples was 10%. These nematodes can be any kind of nematodes including bacteriophore nematodes. However, out of these 15 samples, we were able to re-culture only three nematode isolates from two fields (one isolate from Ron De Yong (Kalispell); two isolates from Mike Leys (Choteau)) (Table 1). These three isolates were considered EPNs as they were able to reproduce in *G. mellonella*. The soils in most of the surveyed areas were alkaline, with soil pH ranging from 6.8 to 8.2. However, the EPN positive isolates were found in soils with a pH of 8.0 to 8.1. The successfully cultured isolates were from sandy clay loam and loam textural classes with organic matter of 3.3 to 3.4% as presented in Table 1. All the successfully cultured EPNs were extracted from wheat fields.

The different morphological characteristics of IJs and males for all the EPN isolates are provided in Tables 2 and 3, respectively. Two isolates collected from Mike Leys (Choteau) were found to be different on the basis of morphological data, resulting in two different isolates from the same field. On the basis of morphological characteristics, the isolate from Ron De Yong (Kalispell) was observed to be from the Steinernematidae. However, one isolate from Mike Leys (Choteau) belongs to Heterorhabditidae while the other is from the Steinernematidae.

**Table 2:** Morphological characters of 3rd stage infective juveniles for three entomopathogenic nematode isolates from Montana, USA.

Character	Steinernema feltiae 1	Steinernema feltiae 2	ANOVA results	Heterorhabditis bacteriophora
L	820.25±11.16 (711-900)^a^	807.85±9.60 (743-906)^a^	*t*=0.82, df=37, *P*=0.11	602.25±9.48 (513-654)
D	28.48±0.73 (22-34.4)^a^	29.93±0.57 (26-35)^a^	*t*=−1.63, df=36, *P*=0.11	24.74±0.64 (18-31)
EP	64.45±1.94 (48-80)^a^	55.25±1.72 (43-67)^b^	*t*=3.45, df=37, *P*=0.001	77.35±2.24 (55-95)
NR	72±2.83 (42-91)^a^	59.65±2.01 (48-81)^b^	*t*=3.47, df=34, *P*=0.001	78.2±1.32 (68-90)
ES	90.2±2.97 (61-114)^a^	87.7±2.02 (48-81)^a^	*t*=0.68, df=33, *P*=0.50	106.5±3.10 (93-140)
T	79.34±1.49 (61-90)^a^	80.85±1.09 (74-89)^a^	*t*=−0.82, df=35, *P*=0.42	91.26±4.52 (56-140.3)
A	29.15±0.81 (24.58-39.68)^a^	27.19±0.6 (23.23-32.78)^a^	*t*=1.90, df=36, *P*=0.07	24.34±0.60 (14.93-28.5)
B	9.31±0.36 (7.45-13.15)^a^	9.31±0.25 (7.40-11.13)^a^	*t*=0.0, df=34, *P*=1.00	5.65±0.18 (3.063-5.52)
C	10.40±0.21 (8.67-13.14)^a^	10.02±0.16 (8.78-11.19)^a^	*t*=1.63, df=37, *P*=0.11	6.60±0.29 (2.10-9.16)
D%	72.42±2.36 (55.24-87.91)^a^	63.62±2.37 (39.81-83.33)^a^	*t*=−1.63, df=36, *P*=0.11	72.63±2.61 (72.29-59.14)
E%	81.60±2.63 (63.16-108.11)^a^	68.42±2.11 (51.81-86.84)^b^	*t*=3.82, df=36, *P*=0.0005	84.75±5.04 (49.52-98.21)

**Notes:** *n*=20. L, body length; D, maximum body diameter; EP, distance from anterior end to excretory pore; NR, distance from anterior end to nerve ring; ES, oesophagus length; T, tail length; A=L/D; B=L/ES; C=L/T; D%=EP/ES ×100; E%=EP/T×100. Figures are showing means ± standard error (μm). For *Steinernema felitae* isolates, means ± SE followed by the same letters are not significantly different at the (*α*=0.05).

**Table 3:** Morphological characteristics of males of three entomopathogenic nematode isolates from Montana, USA.

Character	Steinernema feltiae 1 (1st gen.)	Steinernema feltiae 2 (1st gen.)	*t*-test results	Heterorhabditis bacteriophora (2nd gen.)
L	1570.35±36.84 (1259-1813)^a^	1304.8±43.3 (913-1570)^b^	*t*=4.55, df=37, *P*<0.0001	802.1±20.16 (610-920)
D	96.93±3.01 (76-130)^a^	96.05±3.14 (65-119)^a^	*t*=0.20, df=38, *P*=0.85	62.3±1.99 (50-78)
EP	90.06±2.25 (73-115)^a^	93±1.55 (84-109)^a^	*t*=−1.05, df=34, *P*=0.30	77.9±1.17 (70-90)
NR	98.35±1.63 (88-113)^a^	100±1.18 (88-110)^a^	*t*=−0.80, df=35, *P*=0.43	80.45±1.32 (70-91)
ES	145.55±2.80 (121-167)^b^	164.55±1.72 (154-180)^a^	*t*=−5.63, df=32, *P*<0.0001	110.25±1.97 (97-129)
T	41.65±0.75 (36-48)^b^	52.75±2.62 (34-72)^a^	*t*=−3.96, df=22, *P*=0.0006	61.58±3.59 (41-110)
ABD	44.72±1.07 (35-51)^b^	62.85±3.00 (45-91)^a^	*t*=−5.56, df=24, *P*<0.0001	34.51±0.67 (30-39.4)
SP	80.35±1.41 (67-92)^a^	71.5±2.21 (58-98)^b^	*t*=4.49, df=36, *P*<0.0001	41±1.26 (32-51)
GU	49.95±1.11 (41-59) ^a^	45.85±0.86 (40-53) ^b^	*t*=2.85, df=36, *P*=0.007	22.95±0.74 (14-28)
D%	62.09±1.47 (49.08-72.34)^a^	56.56±0.86 (51.14-65.19)^a^	*t*=0.20, df=38, *P*=0.85	70.90±1.19 (63.2-83.50)
SW%	181.27±4.51 (145.65-235.90)^a^	117.34±6.81 (63.74-168)^a^	*t*=7.63, df=33, *P*<0.0001	119.83±4.69 (91.37-164.52)
GS%	62.74±2.13 (49.40-85.51)^a^	66.35±2.16 (51.19-91.38)^a^	*t*=−1.16, df=38, *P*=0.25	56.85±2.61 (41.18-87.5)

**Notes:** *n*=20. L, body length; D, maximum body diameter; EP, distance from anterior end to excretory pore; NR, distance from anterior end to nerve ring; ES, oesophagus length; T, tail length; ABD, anal body diameter; SP, spicule length; GU, gubernaculum length; D%=EP/ES×100; SW%=SP/ABD×100; GS%=GU/SP×100. For *Steinernema felitae* isolates, means ± SE followed by the same letters are not significantly different at the (*α*=0.05). Figures are showing means ± standard error (μm)

BLASTn analysis showed that the steinernematid recovered from Ron De Yong (Kalispell), and Mike Leys (Choteau) were conspecific to *Steinernema feltiae* (Filipev, 1934). *Steinernema feltiae* recovered from Ron De Yong (Kalispell) and Mike Leys (Choteau) are referred to as isolates *S. feltiae* 1 (GenBank accession: MN647603) and *S. feltiae* 2 (GenBank accession: MN647604), respectively. The two isolates of *S. feltiae* (1 and 2) differed from each other for some morphological characters studied for IJs and males (Tables 2 and 3). In case of IJs, *S. feltiae* 1 had significantly greater distance from anterior end to excretory pore and nerve ring with significantly higher E% (EP/T×100) as compared to *S. feltiae* 2 isolate (Table 2). Similarly, body length, spicule length and gubernaculum length were significantly higher for males of *S. feltiae* 1 as compared to *S. feltiae* 2 isolate (Table 3). However, oesophagus length, tail length and anal body diameter were significantly higher in case of *S. feltiae* 2 males as compared to *S. feltiae* 1 isolate (Table 3).

The second species from Mike Leys (Choteau) was observed to be identical to *Heterorhabditis bacteriophora* (Poinar, 1976) (GenBank accession: MN647605). Overall, the IJs and males of this species were shorter in overall length and other morphological characteristics studied (Tables 2 and 3). In this study, *S. feltiae* 1 was observed to be 100% identical to *S. feltiae* isolates as found in NCBI (GenBank accession: MN044870.1, KT809344.1, KM016378.1, KM016374.1, KM016366.1, KM016361.1, KM016345.1, JN098451.1, AB243439.1). Similarly, *S. feltiae* 2 species was found 100% identical to *S. feltiae* (Accessions: MK294325.1, MK294320.1, KM016352.1, KM016339.1, AF121050). *Heterorhabditis bacteriphora* was 100% conspecific to a number of *H. bacteriophora* isolates in NCBI (MK072810.1, MK421482.1, MG551676.1, KT378450.1, KT378448).

## Discussion

The purpose of this survey was to see if EPNs were present in the Golden Triangle area of Montana, and if so, to explore the patterns of their diversity and distribution. Here, we established the occurrence of native EPNs for the first time in Montana, although the recovery percentage of EPNs was very low. The nematodes present in some soil samples caused *G. mellonella* mortality but were unable to reproduce further. These nematodes might be other rhabditids or opportunistic bacterivore nematodes that feed on saprobic bacteria in the cadaver but cannot reproduce in the cadaver. Different studies were focused toward the co-occurrence and effect of free living, opportunistic bacteriophagous nematodes such as *Oscheius* spp. (Campos-Herrera et al., 2015) and *Pellioditis* sp. (Rhabditida: Rhabditidae) (Duncan et al., 2003) on survival, infectivity and reproduction of EPN species. Duncan et al. (2003) observed the reduced development of *Steinernema riobrave* Cabanillas, Poinar, and Raulston (Rhabditida: Steinernematidae) in the presence of *Oscheius* sp in a laboratory experiment. However, Blanco-Pérez et al. (2017) observed that EPNs were able to reproduce in freeze killed insect cadavers in the presence of scavenger bacteriophagous nematodes (*Oscheius*), though with lower progeny as compared to IJs produced in alive larvae. The reason behind the successful EPN reproduction in this study can be the already freeze-killed insect cadavers. The low recovery rate of nematodes might also be due to the failure of the EPN extraction method (Mráček et al., 2005), unsuitability of the laboratory environment for EPN culture and reproduction (Grewal et al., 1996), or the unsuitability of *G. mellonella* as a host because some EPNs are known to infect only certain hosts (Mráček et al., 2005; Klingen and Haukeland, 2006). In addition, there is a possibility that symbiotic bacterial cells may have been unable to effectively help EPNs reproduce in the hosts. Burnell and Stock (2000) emphasized that a critical number of bacterial cells are required to infect the host insects.

The prevalence of EPNs in agro-ecosystems are largely dependent on a number of biotic including host insects, predators such as mites, parasites, pathogens, free living non-EPNs, etc. and abiotic edaphic factors such as temperature, moisture, and UV light (Lewis et al., 2015; Stuart et al., 2015). Mostly, the fields surveyed in this study were cultivated with dryland farming, and low soil moisture levels might have been one of the most important factors for the absence of EPNs and the low recovery rate. When the level of soil moisture is unfavorable, EPNs can go into a resting phase known as “anhydrobiosis” (Grewal, 2000). The negative effect of pesticides used in these agricultural fields may also be responsible for the absence of EPNs.

The measurements of the morphological characters of the three isolates were observed to be similar to those found by Hominick et al. (1997), with some differences in distance from anterior end to excretory pore, nerve ring and oesophagus for IJs and males. The two isolates of *S. feltiae* differed from each other in respect to spicule length, tail length, anal body diameter, oesophagus, and body length in males and oesophagus in case of IJs. This indicates that despite the genetic similarity between the isolates, there was high morphological variability among isolates. Campos-Herrera et al., (2006) cultured the same Rioja strain of *S. feltiae* (found in Spain) on three insect hosts, *G. mellonella*, *Spodoptera littoralis* Boisd. (Lepidoptera: Noctuidae) and *Bibio hortulanus* L. (Diptera: Bibionidae) and found signiﬁcant differences in morphometric measurements of the IJs developed in different host insects. Similarly, Campos-Herrera and Gutiérrez (2014) also observed the intraspeciﬁc differences in 14 different populations of *S. feltiae* in terms of percentage *G. mellonella* mortality, time to kill the insect, penetration rate with sex-ratio being biased toward females. These morphological differences have been related to the different geographic origin, environmental conditions and host interactions (Stock et al., 2000) and might have been the reasons behind the differences in the morphology of two *S. feltiae* isolates found in the present study. *Steinernema feltiae* in the present study was recovered from two cultivated fields with sandy clay loam and loam textural soil class pH of 8 and 3.3% organic matter. Stock et al. (1999) reported similar results in a survey in California. Similarly, *H. bacteriophora* was found in a wheat field with sandy clay loam texture, pH 8, and 3.4% organic matter. The preference of sandy soils by *H. bacteriophora* has been reported in earlier studies as well (Stock et al., 1999; Campos-Herrera et al., 2012; Stuart et al., 2015). However, Jaffuel et al., (2018) reported different Swiss isolates of *H. bacteriophora* in areas with high clay content and low pH which is in contrast with the previous studies.

*Steinernema feltiae* and *H. bacteriophora* have a near-global distribution and a wide-range of habitats including pastures, roadsides, forests and gardens where human interference is minimal (Hominick et al., 1996; Rosa et al., 2000; Hominick, 2002). Recently, *S. feltiae* and *H. bacteriophora* have been found in Croatia (Majić et al., 2018; Majić et al., 2019). *Steinernema feltiae* can tolerate a wider range of environmental conditions than any other known EPN species. This species has been recovered in fields and grasslands in the UK, the Netherlands, and Germany (Sturhan and Liskova, 1999), and our findings reporting EPN presence from cultivated fields support these results.

Native EPNs already adapted to local environment are thought to be well suited as inundative biological control agents to suppress different insect pests (Shapiro-Ilan et al., 2002, Lewis et al., 2006). These native nematodes can persist longer in the soil, resulting in better biological control efficacy (Koppenhöfer and Fuzy, 2009). More surveys are needed because of the probability of the presence of additional and more virulent EPN species which can be added to the indigenous gene bank for further research. This will increase our understanding of the diversity and biogeography of EPNs. The new species and strains might be utilized in future ecological and biological control studies against different economically important insect pests in Montana as well as other parts of the world with a similar climate.
